# Aneuploidy and Improved Growth Are Coincident but Not Causal in a Yeast Cancer Model

**DOI:** 10.1371/journal.pbio.1000161

**Published:** 2009-07-28

**Authors:** Xin Chenglin Li, John C. Schimenti, Bik K. Tye

**Affiliations:** 1 Department of Molecular Biology and Genetics, College of Agriculture and Life Sciences, Cornell University, Ithaca, New York, United States of America; 2 Department of Biomedical Sciences, College of Veterinary Medicine, Cornell University, Ithaca, New York, United States of America; Dana-Farber Cancer Institute, United States of America

## Abstract

Cancer cells have acquired mutations that alter their growth. Aneuploidy that typify cancer cells are often assumed to contribute to the abnormal growth characteristics. Here we test the idea of a link between aneuploidy and mutations allowing improved growth, using *Saccharomyces cerevisiae* containing a *mcm4* helicase allele that was shown to cause cancer in mice. Yeast bearing this *mcm4* allele are prone to undergoing a “hypermutable phase” characterized by a changing karyotype, ultimately yielding progeny with improved growth properties. When such progeny are returned to a normal karyotype by mating, their improved growth remains. Genetic analysis shows their improved growth is due to mutations in just a few loci. In sum, the effects of the *mcm4* allele in mice are recapitulated in yeast, and the aneuploidy is not required to maintain improved growth.

## Abstract 2

Aneuploidy is a hallmark of cancer cells and is assumed to play a causative role. This relationship is dissected in a yeast, with results that show that anueploidy can be removed, but cells maintain their proliferative advantage.

## Author Summary

Aneuploidy, an abnormality in chromosome number and structure, occurs commonly in cancers and has been suggested to be required to maintain accelerated cell proliferation. However, this hypothesis remains untested as it is not possible to selectively remove the acquired aneuploidy in cells that already have altered growth. Using a yeast model bearing *mcm4^Chaos3^*, an allele that causes mammary tumors in mice, these technical hurdles in animal cells can be overcome. We show that aneuploidy is not responsible for accelerated proliferation in yeast but mutations in just a few loci are. This study provides an excellent example of how a complex disease can be dissected in a simple model organism, and that the information extracted from yeast may be used to guide mammalian studies.

## Introduction

With the sequencing of cancer genome, it has been shown that the tumors of human cancer patients contain numerous genetic alterations [1]. Not all of the alterations promote cancer initiation or progression, the so-called driver events. Recent data indicate that most clonal mutations in tumors lack apparent tumorigenic functions [2]. Among all kinds of alterations, aneuploidy, characterized by changes in chromosomal structure and number, is a remarkably common feature of cancers [3]. It has been proposed that such chromosomal aberrations contribute to characteristics of tumors or precancerous cells through a mechanism by which oncogenes are gained, tumor suppressor genes are lost, or oncogenic fusions are created at breakpoints [4],[5]. However, this proposal had remained untested because of the difficulty of selectively removing the acquired aneuploidy in cells that already have altered growth.

The cancer susceptible allele *Mcm4^Chaos3^* was first identified in a forward genetic mutagenesis screen for mice exhibiting genetic instability (GIN) [6]. MCM4 is a subunit of the evolutionarily conserved heterohexameric MCM2-7 helicase, essential for replication initiation and elongation [7]–[10]. *Mcm4^Chaos3^* (F341I) is located in a conserved region at the interface of neighboring subunits (Figure S1). Female mice homozygous for *Mcm4^Chaos3^* in the C3H strain background are highly prone to aggressive mammary tumors with a mean latency of 12 mo [6]. Most studies on genetic causes of GIN and cancer susceptibility have focused on DNA damage response and cell cycle checkpoint genes rather than the DNA replication machinery. However, there is increasing appreciation that acquired replication stress can be a source of DNA damage that leads to GIN [11],[12]. The *Mcm4^Chaos3^* model is a unique breast carcinogenesis model in that it is not genetically engineered with oncogenes, and it provides an excellent opportunity to investigate the role of DNA replication perturbations on GIN and tumorigenesis.

To understand the effect of *Mcm4^Chaos3^* on genome integrity and its consequences, we introduced the equivalent mutation into diploid yeast. Here, we show that the effect of *Mcm4^Chaos3^* in mice can be recapitulated in yeast. The *mcm4^Chaos3/Chaos3^* diploid yeast shows G2/M delay and severe GIN. We found mutant yeast generate a hypermutable subpopulation that acquires new traits including aneuploidy and improved growth. We took advantage of yeast genetic tools to investigate the link between aneuploidy and mutations that allowed improved growth. We show that neither aneuploidy nor the *mcm4^Chaos3^* mutation contributes to the maintenance of the acquired improved growth phenotype (Igp). Instead, we found that heritable changes unrelated to aneuploidy are responsible for Igp.

## Results

### *mcm4^Chaos3/Chaos3^* Diploid Yeast Exhibit a G2/M Delay

**Figure 1:**
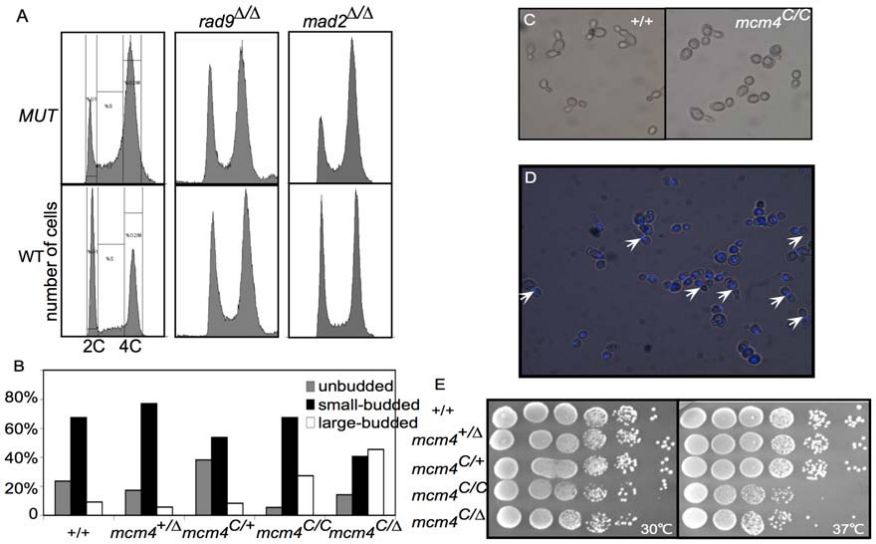
The *mcm4^Chaos3/Chaos3^* mutant has a G2/M delay. (A) The mutant shows a G2/M delay that is Rad9-dependent and Mad2-independent. (B) Homozygous *mcm4^Chaos3^* mutants have a higher mitotic index. Log phase cells were analyzed by microscopy. Cells with no bud (G1), small bud (S), and large bud (G2/M) were counted. (C) Microscopy of log phase *mcm4^Chaos/Chaos3^* and wild-type cells. (D) Fluorescence microscopy of DAPI-stained mutant cells. 77% of mutant large budded cells have one nucleus at the bud neck (pointed with white arrow), whereas 90% of large budded wild-type cells have two nuclei. (E) Serial dilutions of *mcm4^Chaos3^* homozgyotes and hemizygotes grown on YPD at 30°C and 37°C.

We introduced the mouse *Mcm4^Chaos3^* mutation into the orthologous position of *MCM4* (F391I) in diploid yeast [6]. We found that *mcm4^Chaos3/Chaos3^* yeast had a G2/M delay on the basis of FACS analysis of log phase cells (Figures 1A and S2A). At 30°C, the doubling time (DT) of *mcm4^Chaos3/Chaos3^* (3.02±0.16 h) was longer than that of wild-type (2.05±0.06 h) or *mcm4^Chaos3/+^* (2.14±0.06 h) strains. We observed that the proliferating mutant cultures had an increased proportion of large budded cells with one nucleus at the bud neck (Figure 1B–1D), indicating a delay prior to anaphase. This G2/M delay seems to be a checkpoint response triggered by DNA damage. Knocking out the DNA damage checkpoint protein Rad9 [13] abolished the G2/M delay, whereas knocking out the spindle assembly checkpoint protein Mad2 [14] had no effect (Figure 1A). The *mcm4^Chaos3^* allele was slightly temperature-sensitive (ts) for growth (Figure 1E), compared to the reported lethality of other *mcm* mutants at restricted temperature [9]. As in mice [6], these defects are more severe in the yeast *mcm4^Chaos3^* ^/Δ^ mutant (Figures 1B, 1E, and S2A), which has a DT of 3.72±0.15 h. The growth defects in *mcm4^Chaos3/Chaos3^* is partially rescued by one copy of the wild-type *MCM4* (Figure S2B) with a DT of 2.28±0.13 h, while no further increase of DT was observed in wild-type strain with an additional copy of wild-type *MCM4* (2.00±0.02 h).

### The *mcm4^Chaos3/Chaos3^* Diploid Shows a 100-Fold Increase in Loss of Heterozygosity Because of Hyperrecombination

Loss of heterozygosity (LOH) is a major contributing event in cancer development and a product of GIN. To investigate whether the *mcm4^Chaos3^* allele causes GIN in yeast, we measured the LOH frequency of *CAN1* with respect to *HOM3* on the left arm of chromosome V [15]. Almost all detected LOH events were due to mitotic recombination. There was little difference in the frequency between *MCM4^+/+^* (2.12±0.11×10^−5^) and *mcm4^Chaos3/+^* (3.04±0.73×10^−5^) yeast, but the frequency in *mcm4^Chaos3/Chaos3^* (2.60±1.60×10^−3^) was about 100-fold elevated over that of the wild type. This frequency is much higher than any DNA damage checkpoint, recombination, or repair mutants reported to date [16],[17].

### A Subpopulation of *mcm4^Chaos3^* Cells Form Colonies Slowly

**Figure 2:**
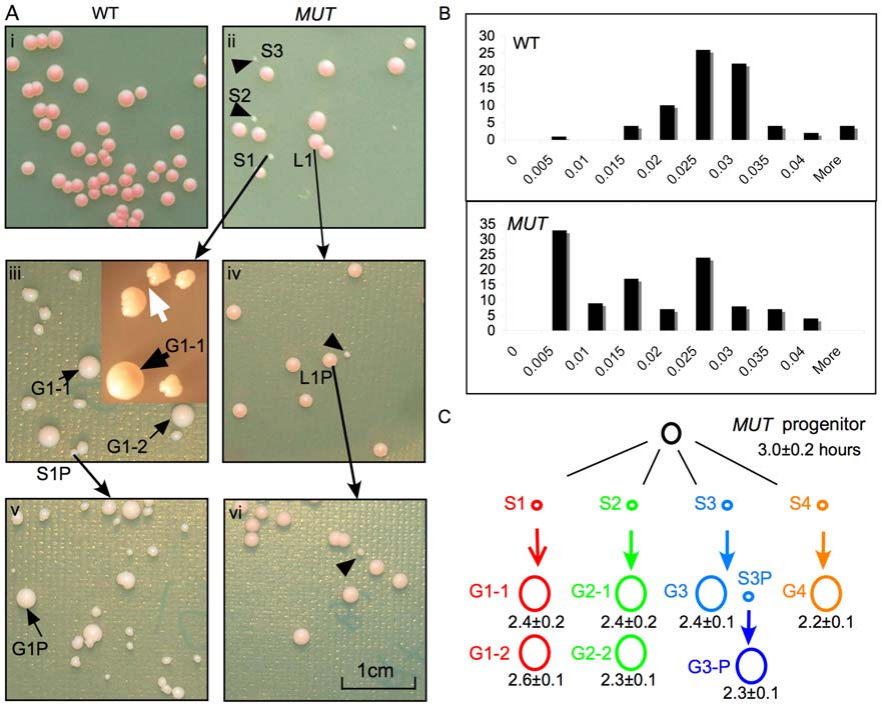
The *mcm4^Chaos3/Chaos3^* mutant generates a subpopulation of genetically unstable cells. (A) The mutant produces heterogeneous offspring. (i) Wild-type cells produce uniform sized colonies. (ii) The mutant produces heterogeneous sized colonies. The arrowheads point at representative minute colonies, S1, S2, and S3. Long black arrows indicate the lineage of colonies that were analyzed. L1 and S1 are a large and a minute colony derived from a streak of a large colony of the mutant. (iii) and (iv) are colonies derived from S1 and L1, respectively. (iii) Heterogeneous colony morphologies include giant (G1-1, G1-2), serrated (white arrow), and minute (S1P) colonies. The inset is a magnification of the heterogeneous colonies. (v) S1P gives rise to heterogeneous colonies including giant colonies such as G1P. (vi) Large colonies (L1P) consistently give rise to both large and minute colonies. Scale bar of 1 cm is shown. (B) Histograms of colony size of wild type (normal distribution) and *mcm4^Chaos3/Chaos3^* (bimodal distribution). (C) The lineage of strains presented in Figures 2 and 3. Ancestral progenitor is represented by a black circle. Different minute colonies are color coded. Giant colonies derived from the same ancestral minute colony are coded with the same color. The number under each strain is the DT (h). S, minute; L, large; G, giant; P, progeny.

*mcm4^Chaos3/Chaos3^* yeast cultures showed 40% decreased viability (Figure S2C) compared to wild type and gave rise to a subpopulation that formed minute colonies (Figure 2A, ii). Whereas colonies of wild-type yeast are uniform in size, we found that mutant yeast formed variably sized colonies with a bimodal distribution (Figure 2B). This bimodal distribution of large and minute colonies was reproduced upon replating of the large colonies (Figure 2A, ii, L1 and 2A, iv, L1P). Replating of the minute colonies gave rise to a dramatically heterogeneous distribution (Figure 2A, iii), including minute, serrated (white arrow), and giant colonies (G1-1 and G1-2). The minute S1P retained the ability to produce heterogeneous offspring including giant colonies (Figure 1B, v, G1P) upon restreaking. The serrated morphology is typical of yeast cells that are continuously giving rise to offspring with different viabilities and growth rates [18]. A key observation is that giant colonies readily emerge from a single restreaking of minute colonies, but rarely from the direct restreaking of large colonies as if an intermediate step (which we hypothesize to involve hypermutagenesis) is required for this transition.

### Progeny of Minute Colonies Acquire New Traits

The giant colonies were interesting to us because of their size and smooth morphology, traits indicative of cells having a relatively shorter DT and more stable genome than their progenitors that form the minute colonies (minute progenitors). An obvious explanation for their emergence is that secondary genetic events must have overcome the growth defects of the minute progenitors. To investigate these secondary genetic events, seven giant colonies with lineages traced to a single founder cell were characterized (Figure 2C). All growth measurements are referenced against that of the ancestral *mcm4^Chaos3/Chaos3^* progenitor that generates both large and minute colonies because the minute progenitors are severely unstable. Consistent with their colony size, cells forming giant colonies had shorter DTs than their ancestral progenitor (Figure 2C) and proliferated much faster than their minute progenitors.

**Figure 3:**
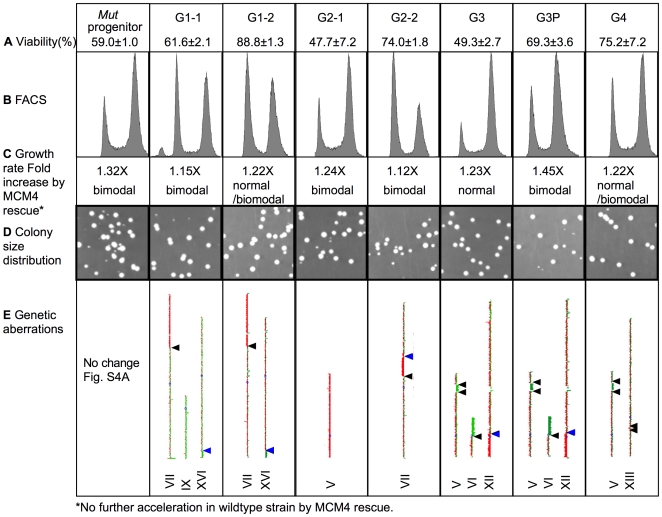
New traits acquired by cells of giant colonies. Viability (A), FACS profiles (B), fold increase in growth rates, with or without wild-type *MCM4* on a *CEN* plasmid (C), colony size distribution (D), and aneuploidy (E). Only affected chromosomes are shown in (E): yellow indicates approximately equal amounts of hybridization between mutant and wild-type DNA; green indicates approximately 2-fold reduction; and red approximately 1.5–2-fold increase in mutant. Arrowheads (black, Ty; blue, solo LTR) represent the breakpoints of translocations, amplifications, or deletions. Detailed characterization of colony size distributions and genetic aberrations are shown in Figures S2D and S3, respectively.

Other than the common Igp, each strain exhibited additional distinct new traits. Some have viability that surpasses that of the ancestral progenitor, while some have decreased viability (Figure 3A). FACS analysis indicated that these strains still maintained a near-diploid DNA content, and some of them had a less pronounced G2/M delay than their ancestral progenitor (Figure 3B). The distribution of colony size also varied among these strains (Figures 3D and S2D). Some of them became sensitive to genotoxic drugs such as hydroxyurea (Figure S2E). The distinct new traits of the giant colony-forming cells suggest that these traits are acquired independently and that the Igp of independent giant colonies may result from different underlying mechanisms.

To investigate whether maintenance of the improved growth state requires *mcm4^Chaos3^*, we complemented the *mcm4^Chaos3^* mutation by transforming a wild-type *MCM4* allele into these fast-proliferating strains. We have shown earlier in Results (Figures 1 and S2A) that phenotypes such as reduced viability, hyperrecombination, and G2/M delay caused by *mcm4^Chaos3^* are recessive. If, in addition to the secondary mutations, *mcm4^Chaos3^* is required for improved growth, the presence of a wild-type *MCM4* allele would slow down the growth. However, proliferation rates of the fast-proliferating strains (Figure 3C) were further accelerated by *MCM4*, suggesting that some other genetic events are responsible for the Igp independent of the *mcm4^Chaos3^* background. This result also suggests that the newly acquired mutations are not merely *mcm4^Chaos3^* specific suppressors. Thus, unlike oncogene-induced proliferation [19], the *mcm4^Chaos3^* mutation that initiates GIN is not required to maintain the improved growth state.

### Fast-Proliferating *mcm4^Chaos3/Chaos3^* Strains Are Associated with Various Types of Genetic Alterations

To investigate the effects of *mcm4^Chaos3^* on genome integrity and the genetic events associated with Igp, we analyzed the karyotypes of these seven fast-proliferating strains by array-CGH and, when translocations were apparent, by PCR and pulse field gel electrophoresis. Each strain had a unique spectrum of aneuploidy or chromosomal aberrations, including translocations, segmental duplications and deletions, whole chromosome gains or losses, and gene amplifications (Figure 3E). The perfect correlation between Igp and aneuploidy in these seven randomly selected large colonies was striking. However, we did not observe a common chromosomal aberration that could be identified as a defining primary genetic change responsible for the Igp. We found that the breakpoints of all of the chromosomal rearrangements were associated either with Ty or solo long terminal repeat (LTR) elements (Figures 3E, arrowheads, and S3). Tys and LTRs have been shown to be hotspots for translocation [20]–[22].

### Aneuploidy Is Not Responsible for Improved Growth

**Figure 4:**
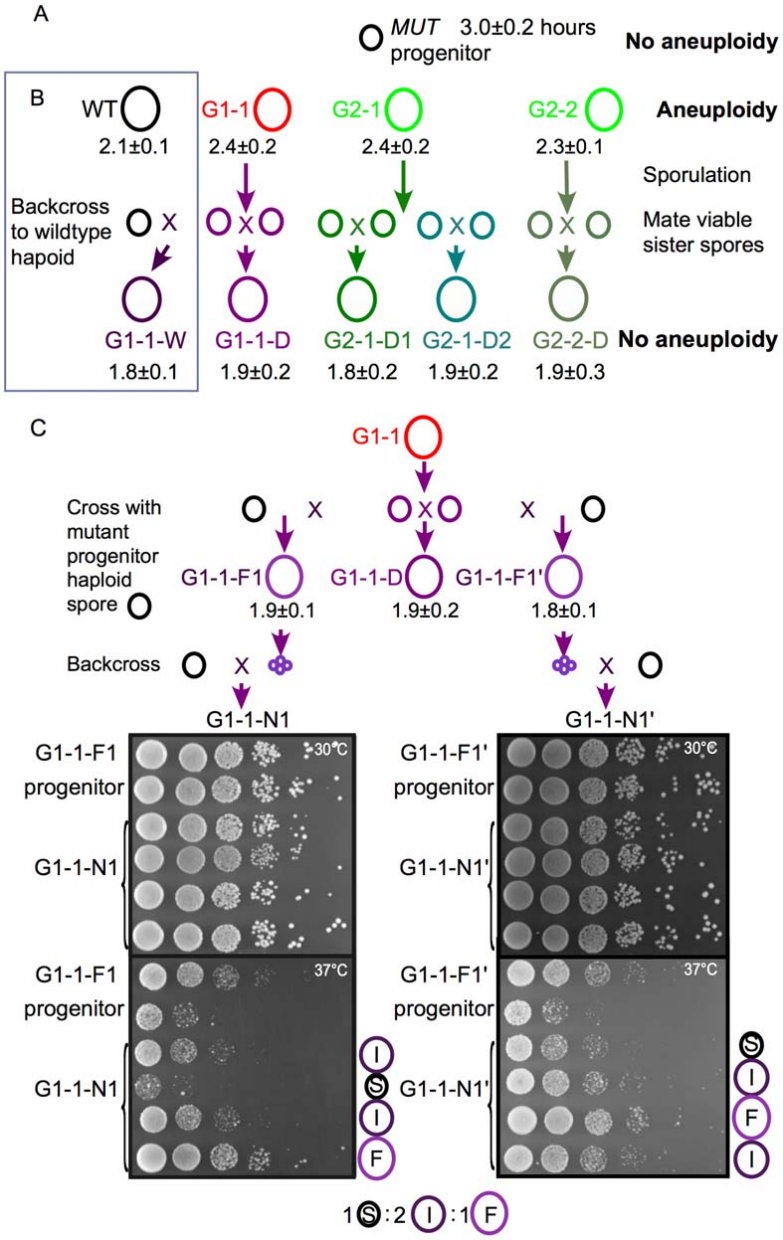
Mutations unrelated to aneuploidy contribute to improved growth. (A) Cells from giant colonies were sporulated and sister spores were mated. Those with Igp were devoid of aneuploidy (confirmed by CGH, see Figure S4) and show even more enhanced proliferation rates. DTs of the resulting diploids are shown. Spores with the same color are from the same tetrad. (B) A parent of G1-1-D was crossed with wild-type haploid to generate G1-1-W. (C) The Igp of G1-1-D is dominant and segregates 1∶2∶1 in tetrads. The parents of G1-1-D were crossed with progenitor *mcm4^Chaos3^* strain to form G1-1-F1 and G1-1-F1′, which were sporulated for tetrad analysis. Tetrads were backcrossed to the progenitor *mcm4^Chaos3^* strain (colored black) for Igp. The growth rates of the resulting diploids, G1-1-N1 and G1-1-N1′ were compared by plating on YPD plate at 30°C and 37°C. An additional tetrad generated from G1-1-F1 is shown in Figure S4F. F, fast; I, intermediate; S, slow.

The perfect correlation between aneuploidy and Igp suggests a causal relationship. To investigate the causative effect of aneuploidy on improved growth, we removed chromosomal aberrations from the fast-proliferating strains by sporulating G1-1, G2-1, G2-2, and mated sister spores (Figure 4A). We then performed CGH on the derivative diploids to verify the presence or absence of chromosomal aberrations. G1-1D, G2-1D-1, G2-1D-2, and G2-2D showed no aneuploidy (Figure S4) but all exhibited even shorter DTs than their giant parent strains (Figure 4A). This result suggests that CGH-detectable aneuploidy is not required for Igp. Rather, other secondary mutations or epigenetic alterations contribute to Igp. The tight correlation between aneuploidy and Igp without a demonstrable causal relationship suggests that these traits co-emerge from the same process, presumably involving a hypermutable slow phase driven by *mcm4^Chaos3^* that allows for the acute accumulation of a large number of genetic alterations within a short period of time.

Another approach to investigate whether and which specific chromosome aberration may be responsible for Igp is to correlate specific aneuploidy and proliferation based on lineage. If aneuploidy were associated with Igp, slow-growing siblings of fast growers would not be aneuploid or would have distinct genetic aberrations. The giant colony G3's minute sibling (S3P) was streaked further to generate G3P because S3P was too unstable for karyotype analysis (Figure 2C). Remarkably, we found that G3 and G3P share multiple identical genetic aberrations (Figure 3E). These aberrations unlikely arose independently and more likely arose in S3, the slowly proliferating minute progenitor cells of G3 and S3P. Therefore the progenitor cell of S3P must have already acquired the aneuploidy that is associated with improved growth in G3 and G3P, suggesting that aneuploidy is unrelated to Igp. Despite their identical aneuploidy, G3 and G3P have distinctly different viability, cell cycle profiles, and colony sizes (Figure 3, Figure S2B). Such traits presumably are caused by genetic changes distinct from the shared chromosome alterations and were acquired independently during clonal expansion of their respective minute progenitors.

### Mutations Responsible for the Igp Segregate in a Mendelian Fashion

We have shown that aneuploidy is not the cause of the Igp. So what events cause Igp? Is it possible to genetically map the loci in these cells? The parents of the fast-proliferating strain (G1-1D) were backcrossed with the ancestral progenitor *mcm4^Chaos3^* strain (Figure 4C) that does not have secondary mutations. The resulting diploids (G1-1-F1 and G1-1-F1′) in a heterozygous background for the secondary mutations also show improved growth (Figure 4C), indicating that the Igp in G1-1D is dominant. Mating the G1-1D spore with wild-type haploid results in further improved growth (DT = 1.8±0.1 h compared to wild-type DT = 2.1±0.1 h) (Figure 4B). To test whether the Igp is due to epigenetic modifications such as histone H3 and H4 lysine deacetylation, we treated wild-type G1-1-F1 and G1-1-N1 with histone deacetylase inhibitors: the histone deacetylase inhibitors nicotinamide (NAM) and Trichostatin A (TSA), repressing nicotinamide adenine dinucleotide (NAD)-dependent and class I or II histone deacetylases, respectively [23],[24]. Our results showed that in vivo treatment with NAM and TSA had no effect on Igp (Figure S4G), suggesting that the Igp in G1-1 is due to genetic mutations rather than epigenetic modifications. If the Igp is dominant and if it is determined by no more than one or two alleles, one should be able to observe Mendelian segregation of the mutation(s) linked to Igp by tetrad analysis. G1-1-F1 and G1-1-F1′ were sporulated. Three tetrads of G1-1-F1 and G1-1-F1′ were mated to the progenitor *mcm4^Chaos3^* strain to further analyze the proliferation proficiency. Instead of measuring growth rates at 30°C, the segregation pattern of the Igp was best demonstrated by plating the resulting diploids on yeast peptone dextrose (YPD) plates at 37°C. The Igp segregated 1∶2∶1 in all three tetrads examined (Figures 4C, S4F, and S4G) suggesting that two alleles in G1-1-F1 and G1-1-F1′ constituted the Igp. We do not know if these alleles are identical for G1-1-F1 and G1-1-F1′. If so, the parents of G1-1D are parental ditypes caused by independent assortment of two mutations or LOH may have played a role in the homozygosity of these alleles in G1-1. This genetic approach may be applied to individual fast-proliferating strains to estimate the number of alleles that contribute to the Igp.

### The Subpopulation Forming Minute Colonies Is Hypermutable

The ancestral progenitor does not harbor any aneuploidy (Figure S4A), so the aneuploidy in the fast-proliferating strains must be acquired during formation of minute colonies. To investigate when aneuploidy was acquired, we compared the karyotypes of pairs of fast-proliferating strains each derived from a common minute progenitor. Giant colonies G1-1 and G1-2, both derived from minute colony S1 (Figure 2C), shared a common translocation of a segment of the right arm of Chromosome VII to the left arm of Chromosome XVI (Figures 3E and S3A), suggesting that this particular translocation event may have occurred very early during the clonal expansion of S1. However, G1-1 also had a loss of Chromosome IX, an event not shared by G1-2, suggesting that Chromosome IX was lost later during the clonal expansion. This result suggests that the subpopulation of *mcm4^Chaos3/Chaos3^* cells that form minute colonies are genetically unstable, a property that is consistent with the heterogeneous morphologies of colonies generated by these cells upon restreaking.

The comparison of G2-1 and G2-2 also indicates that aneuploidy is acquired during the clonal expansion of S2. G2-1 and G2-2 shared no common gross chromosomal aberration (Figures 3E and S3B), suggesting that the gain of Chromosome V in G2-1 and the segmental duplication of Chromosome VII must have been generated late after the emergence of the S2 progenitor cell. We estimate that ∼20 cell divisions are required to form a visible colony of 10^6^ cells in a minute colony. In both of these examples, independent gross chromosome rearrangements took place late during the clonal expansion of S1 and S2 within a short period of fewer than 20 cell divisions from the birth of the founder cell.

### The Hypermutable Slow Phase Is Critical for the Rapid Emergence of Improved Growth Traits

**Figure 5:**
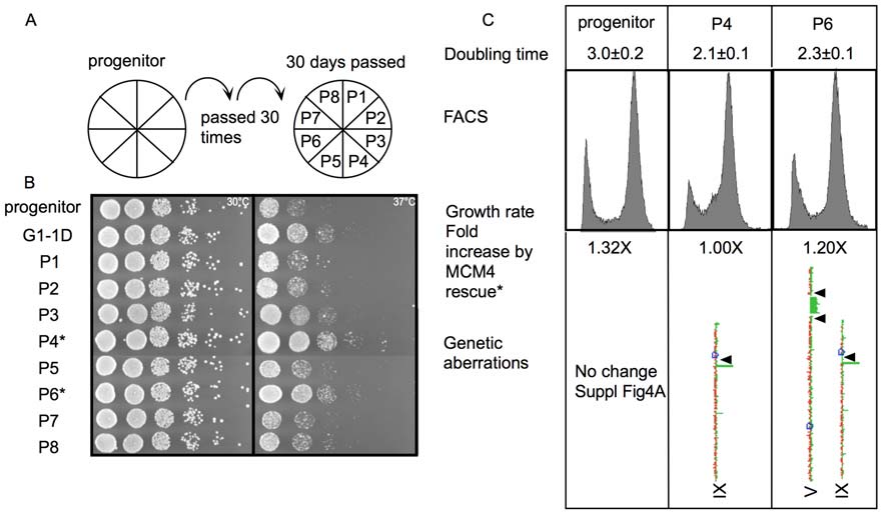
The evolution of *mcm4^Chaos3^* in the main population. (A) The scheme of independent serial passages of *mcm4^Chaos3^* progenitor on YPD. A swipe of cells was streaked out on YPD plates every day for 30 d. (B) Serial dilutions of *mcm4^Chaos3^* strains after 30 passages grown on YPD at 30°C and 37°C. P4 and P6 showing obvious Igp are labeled with asterisks. (C) The DT, FACS profile, fold increase in growth rates, with or without wild-type *MCM4*, on a *CEN* plasmid and karyotype of P4 and P6. Arrowheads (black, Ty) represent the breakpoints of deletions, and regions of gene loss (Figure S5).

All of the fast-proliferating strains so far were derived from cells that form minute colonies. We did not observe giant colonies from the direct streaking of large colonies presumably because hypermutable cells have a severe growth disadvantage in the main population and the emergence of Igp requires the gradual accumulation of mutations through successive hypermutagenic cell divisions. To investigate whether the main population will allow the emergence of Igp, a swipe of cells from eight independent large colonies was patched on YPD plate and then repatched on a fresh plate daily for 30 d in a “chemostat on plate” experiment (Figure 5A). After 30 d and approximately 300 cell divisions, we assayed each of the eight independent cell lines for Igp. We found two of the eight or 25% of the cell lines have acquired an Igp (Figure 5B, P4 and P6) in contrast to the emergence of Igp in 100% of the minute colonies analyzed. This result suggests that the subpopulation that forms minute colonies is hypermutable compared to the main population, and that propagation of hypermutable cells free from the main population is critical for the rapid generation of Igp in the *mcm4^Chaos3^* mutant.

Both cell lines, P4 and P6, are homozygous for the same gene deletion Δ(*VID28-SNL1-BAR1*) on Chromosome IX (Figure S5), and P6 contains an additional segmental deletion on Chromosome V (Figure 5C), identical to that found in G3, G3P, and G4. Mating viable spores removed the segmental deletion in P6 (DT = 2.3±0.1 h) to generate P6-D (1.9±0.1 h), confirming again that this segmental deletion is unlinked to the Igp. Introducing a wild-type copy of *MCM4* into P4 and P6 did not impede the proliferation rate indicating that *mcm4^Chaos3^* mutation is not required to maintain the Igp (Figure 5C). This result suggests that independent of the pathway of achieving improved growth, whether through a hypermutable subpopulation within a short period of time or through gradual adaptations in the main population, the simultaneously acquired aneuploidy and the *mcm4^Chaos3^* mutation are not responsible for the Igp.

## Discussion

### The Effects of Mcm4^Chaos3^ in Mice Are Recapitulated in Yeast

In this study, we have shown that a mutation in *MCM4* that predisposes mice to mammary adenocarcinomas also predisposes yeast to improved growth. There are other striking similarities between the mouse and yeast mutant such as elevated GIN, G2/M delay, and chromosomal abnormalities (Table 1). A subtle defect in the MCM helicase that had little deleterious effect on the whole animal in mice or the main cell population in yeast somehow acts as a driving force to create aneuploidy in a subpopulation of cells. The locations of the breakpoints of the chromosomal rearrangements at Ty and solo LTR elements suggest that replication fork defects either occur or are repaired at these sites. Further investigation should provide insight into the molecular events at the replication fork that induce the hypermutable phase that is so vividly manifested in the minute colonies in this study. This study provides an excellent example of the utility of yeast as a simple model organism for dissecting the molecular basis of complex diseases. Information extracted from yeast about altered pathways or genes that enhance cell proliferation may be used to guide mammalian studies.

**Table 1:** Phenotypic similarities between *mcm4^Chaos3^* yeast and *Mcm4^Chaos3^* mice.

Yeast	Mice^a^
G2/M delay	G2/M delay in *Mcm4^C/C^* MEFs and developmental lethality in *Mcm4^C^* ^/Δ^ mice
Translocation and segmental deletion or amplification at LTR sites.	Embryonic fibroblasts highly susceptible to chromosome breaks under replication stress
100-fold increase in mitotic recombination	20-fold increase in frequency of micronuclei in erythrocytes, likely representative of elevated DSBs
Predisposition to improved growth	80% of females acquire aggressive mammary tumors
Particular chromosome abnormalities in individual improved growth strains	Different segmental aneuploidies in independent tumor cell lines (detected by array CGH; unpublished results)

a See [6],[41].

### Aneuploidy and Improved Growth That Co-Emerge as New Traits Are Unlinked

Concerted efforts to sequence breast cancer genomes to identify the genomic changes that cause breast cancers have been launched both in the US and in the UK [3],[25],[26]. Preliminary analysis of 24 breast cancers reveals that as many as 2,000 rearrangements associated with these representative subclasses of breast cancer; deep sequencing of a couple of other cancers indicates thousands of point mutations in each cancer (M. Stratton, personal correspondence). Identification of the driver mutations responsible for breast cancer among this vast number of passenger mutations is daunting indeed. Relevant simple models, such as the *mcm4^Chaos3^* yeast mutant, are needed to provide insight for sorting out driver from passenger mutations in the human cancer genome studies.

The 100% coincidence of aneuploidy and improved growth (see correlation calculation in Materials and Methods) in seven randomly selected fast-proliferating strains in this study provides a perfect test for the hypothesis that aneuploidy and Igp are linked in cancer cells. Using two different approaches, we demonstrated that aneuploidy is unlinked to Igp. First, we removed aneuploidy from fast-proliferating cells by genetic crosses and showed that cells stripped of aneuploidy have further improved growth. Second, we delineated the phylogeny of subclones derived during clonal expansion from an ancestral *mcm4^Chaos3^* cell and showed that siblings harboring identical aneuploidy have dramatically different growth rates. Both of these approaches are unique to the yeast model because in animal studies for cancer development, it is not possible to trace the ancestral cell with the initiating oncogenic mutation in a tumor [27] or to remove aneuploidy from cancer cells without introducing additional genetic alterations. Our results complement two recent yeast and mouse studies that show that artificially constructed strains or primary cells bearing an extra copy of a chromosome does not lead to improved growth [28],[29]. Our study addressed the role of aneuploidy in the later stage when the cells already acquired altered growth and chromosome aberrations, demonstrating that naturally acquired aneuploidy is not required to maintain the improved growth traits. Importantly, our study was not limited to chromosome gains, but other spontaneous chromosomal aberrations associated with improved growth such as chromosome loss, translocations, segmental duplications, and deletions.

### Mutations That Improve Cell Proliferation

If aneuploidy is not responsible for the Igp of any of the fast-proliferating cells that we randomly selected, what are the mutations responsible? We sporulated the fast growing diploids and backcrossed to the progenitor strain and then carried out tetrad analysis. We showed that the G1-1 strain is dominant for the Igp and the mutant alleles segregated 1∶2∶1. This segregation pattern is unchanged by treatment with NAM or TSA (Figure S4G), suggesting that two unlinked mutations act independently to improve growth (Figure 4C). G2-2 on the other hand is recessive for the Igp (unpublished data). The important point here is that we believe that many mutations that cause Igp can be identified. We speculate that fast growers with recessive mutations might include mutants compromised in checkpoint defects that shorten the cell division cycle, whereas dominant mutations might include metabolic mutations that increase energy production or gain of function mutations such as those found in p53 in mammals [30]. The identification of these mutations might provide insight into the many causes of uncontrolled cell proliferation that is characteristic of cancer cells.

### A Hypermutable Slow Phase Is an Intermediary State for the Rapid Emergence of New Traits

The bimodal colony size distribution is a unique feature of the *mcm4^Chaos3^* diploid mutant. Although the main population of *mcm4^Chaos3^* diploid displays a G2/M delay, a 100-fold increase of LOH, and a subtle growth defect, the subpopulation that forms minute colonies has acute phenotypes. The hypermutable property of this subpopulation most likely contributed to the reduced viability of the population as a whole.

The classical view for the relationship between GIN and cancer is that only cells with subtle GIN undergo tumorigenesis by incremental adaptations [31] because cells with severe GIN are eliminated by apoptosis or survival pressure. In this study, we find that the hypermutable cells with severely compromised growth are the ones that ultimately generate fast growers when given the opportune environment to propagate without survival pressure. This observation suggests that GIN alone in the absence of survival pressure is sufficient to generate fast growers. In contrast, within the main cell population where survival pressure weeds out the hypermutable cells that have a growth disadvantage, the process of acquiring new traits such as Igp is less effective (Figure 5). As a result, the main population of *mcm4^Chaos3^* progenitor undergoes apparent self-renewal for generations without dramatic changes of its characteristics. Another view for the relationship between GIN and cancer is that a loss of checkpoint control allows the survival of hypermutable cells [11],[12], which might be important during the formation of the minute colonies.

The existence of a hypermutable slow phase with severe growth defects during the development of fast-proliferating cells reconciles with many of the concepts that emerge from the debate about the cause and effect of GIN. Although GIN alone is deleterious [32], given a situation when survival pressure is alleviated, cells with GIN are able to quickly accumulate a large number of mutations, and beneficial mutations among them eventually overcome the deleterious effects of GIN. Such a hypermutable slow stage that escapes survival pressure has been hypothesized to exist in early tumorigenesis [33],[34]. Our study provides direct evidence for the existence and importance of such a hypermutable slow stage for the adaptation of cells that ultimately achieve a high proliferative capacity.

## Materials and Methods

### Yeast Strains and Media

Isogenic diploid W303 yeast strains *mcm4^+/+^*, *mcm4^+/Chaos3^*, *mcm4^+^* ^/Δ^, *mcm4^Chaos3/Chaos3^*, and *mcm4^Chaos3^* ^/Δ^ were constructed as described [6]. Strains used in this study are listed in Table S1. Histone deacetylase inhibitors were added to YPD media at 5 mM for NAM (Sigma) or 10 µM for TSA (Sigma).

### Flow Cytometric Analysis

Approximately 1×10^7^ cells were collected from log-phase cultures and processed as described [35]. DNA was stained with Sytox Green (Molecular Probes) and profiles were analyzed using a Becton Dickinson LSR II with a 530/30BP channel filter and BDFACSDiVa software Becton Dickinson.

### Growth Curve and DT

Saturated cell cultures were diluted 25× in complete medium and then grown at 30°C for 4 h to mid-log phase. The absorbance at 600 nm was measured every half hour for 5 h. The growth rates and DTs were calculated during exponential growth. For each experiment where DTs of different strains are compared, all strains were processed simultaneously in at least two independent trials to yield variations in DTs of less than 0.1 hr. Relative differences in DT were confirmed using microplate reader Tecan Infinite M200.

### Cell Viability and Colony Size Distribution

Cell viabilities were measured by first counting log phase cells in a hemacytometer before plating in triplicate on YEPD and counting visible colonies after 3 d of growth at permissive temperatures. Colony sizes were quantified by ImageJ, and histograms were plotted by Excel.

### Mitotic Recombination Assay

A standard assay for measuring mitotic recombination and chromosome loss was used [15]. The test strain was heterozygous for mutations in *CAN1* and *HOM3*, two markers located on opposite arms of Chromosome V. The haploid strain with the *can1* mutation was resistant to canavanine (Can^r^) and the *hom3* strain was auxotrophic for threonine (Thr^−^). Heterozygous diploid strains were Can^s^ and Thr^+^. Mitotic recombination was scored by the Can^r^ Thr^+^ phenotype. Over 90% of the Can^r^ strains scored were Thr^+^.

### Comparative Genomic Hybridization Microarray

Genomic DNA was prepared, sonicated, and labeled on the basis of the protocol from the Dunham lab [28]. DNA from the experimental strain was labeled with Cy3 nucleotide, and DNA from wild-type strain was labeled with a Cy5 nucleotide. The two DNA samples were mixed and hybridized to Yeast Whole Genome ChIP-on-chip Microarray from Agilent (290 nt resolution, 4×44 K-slide format, which contains ∼85% of the nonrepetitive portion of the yeast genome catalog number G4493A). Arrays were then washed according to the Agilent SSPE wash protocol, and scanned on an Agilent scanner or Axon 4000B microarray scanner. The image was processed using the default settings with Agilent Feature Extraction software or GenePix Pro 6.0. All data analysis was performed using the resulting log2 ratio data, and filtered for signals that are 2.5-fold above background in at least one channel. Chromosome translocations are confirmed by PCR analysis and pulsed field electrophoresis.

### Correlation between Improved Growth and Aneuploidy

The confidence level of the correlation between improved growth and aneuploidy based on seven randomly selected giant colonies is 1−*n* ^7^ where *n* is the probability of chromosome rearrangement occurring in a single cell. Chromosome rearrangement is a rare event generated by mitotic recombination, which occurs at a frequency of ∼1×10^−3^ in the *mcm4^Chaos3^* diploid. Thus, if *n* is <1×10^−3^, then the confidence level is close to 1 and the correlation is 100%.

## Supporting Information

Figure S1 The mouse *Chaos3* mutation F345I is located in a conserved region of MCM4 at the interface between subunits [36],[37]. (2.17 MB PDF)

Figure S2 The traits of the progenitors and fast-proliferation strains [37]. (2.84 MB PDF)

Figure S3 Chromosomal features around the breakpoints of genetic aberrations shown in Figure 2E [38]–[40]. (4.33 MB PDF)

Figure S4 Improved growth strains derived by outcrossing the chromosome aberrations. (3.31 MB PDF)

Figure S5 Chromosomal Features around the gene loss sites of P4 and P6 shown in Figure 5C. (0.55 MB PDF)

Table S1 Strain list. (0.07 MB DOC)

## Abbreviations

DT: doubling time
GIN: genetic instability
Igp: improved growth phenotype
LOH: loss of heterozygosity
LTR: long terminal repeat
NAM: nicotinamide
TSA: Trichostatin A
YPD: yeast peptone dextrose
