# Supplementary material for: Aneuploidy and Improved Growth Are Coincident but Not Causal in a Yeast Cancer Model
Source: PLoS Biol. 2009 Jul 28;7(7):e1000161. doi: 10.1371/journal.pbio.1000161 (PMC2708349; doi:10.1371/journal.pbio.1000161)
Supplement: Figure S1 — (2.17 MB PDF) [file pbio.1000161.s001.pdf]

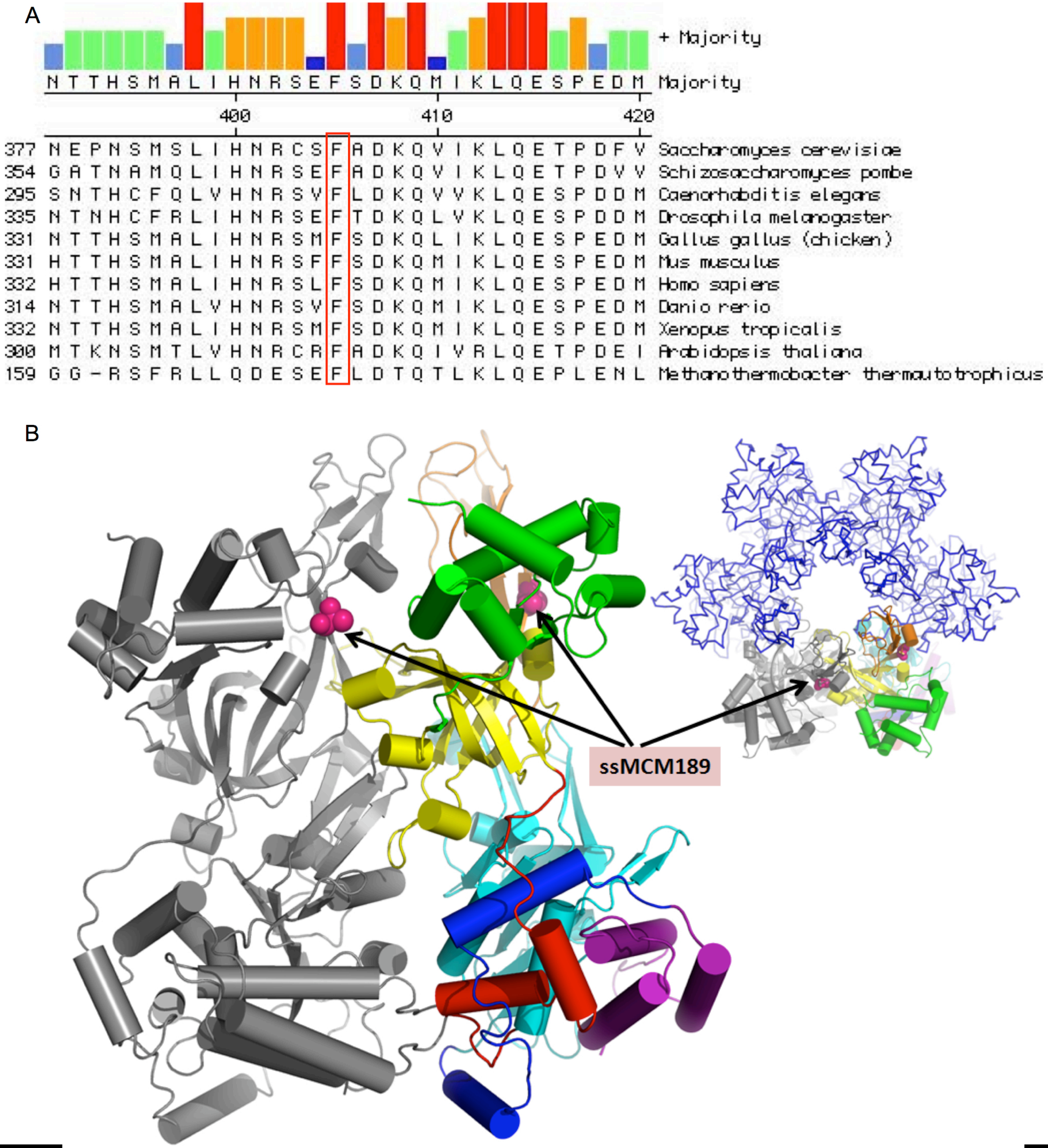
A
+ Majority
N T T H S M A L I H N R S E F S D K Q M I K L Q E S P E D M Majority
400
410
420
377 N E P N S M S L I H N R C S F A D K Q V I K L Q E T P D F V Saccharomyces cerevisiae
354 G A T N A M Q L I H N R S E F A D K Q V I K L Q E T P D V V Schizosaccharomyces pombe
295 S N T H C F Q L V H N R S V F L D K Q V V K L Q E S P D D M Caenorhabditis elegans
335 N T N H C F R L I H N R S E F T D K Q L V K L Q E S P D D M Drosophila melanogaster
331 N T T H S M A L I H N R S M F S D K Q L I K L Q E S P E D M Gallus gallus (chicken)
331 H T T H S M A L I H N R S F F S D K Q M I K L Q E S P E D M Mus musculus
332 H T T H S M A L I H N R S L F S D K Q M I K L Q E S P E D M Homo sapiens
314 N T T H S M A L V H N R S V F S D K Q M I K L Q E S P E D M Danio rerio
332 N T T H S M A L I H N R S M F S D K Q M I K L Q E S P E D M Xenopus tropicalis
300 M T K N S M T L V H N R C R F A D K Q I V R L Q E T P D E I Arabidopsis thaliana
159 G G - R S F R L L Q D E S E F L D T Q T L K L Q E P L E N L Methanothermobacter thermautotrophicus
B
ssMCM189
