# Supplementary figures and images for: Aneuploidy and Improved Growth Are Coincident but Not Causal in a Yeast Cancer Model
Source: PLoS Biol. 2009 Jul 28;7(7):e1000161. doi: 10.1371/journal.pbio.1000161 (PMC2708349; doi:10.1371/journal.pbio.1000161)

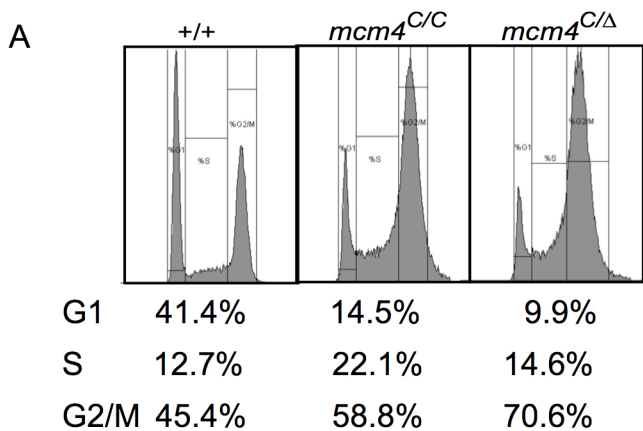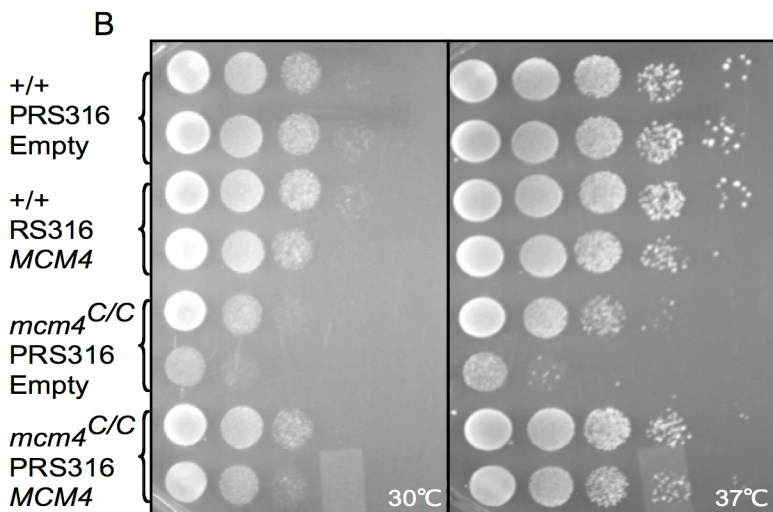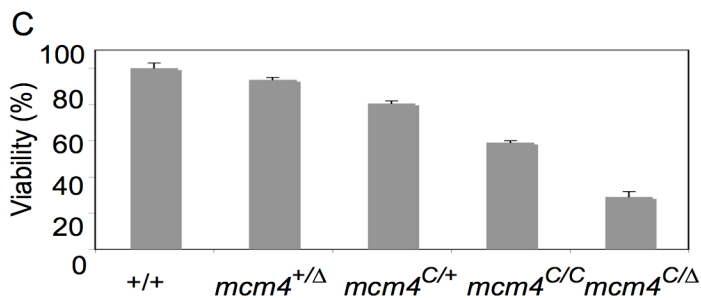

D

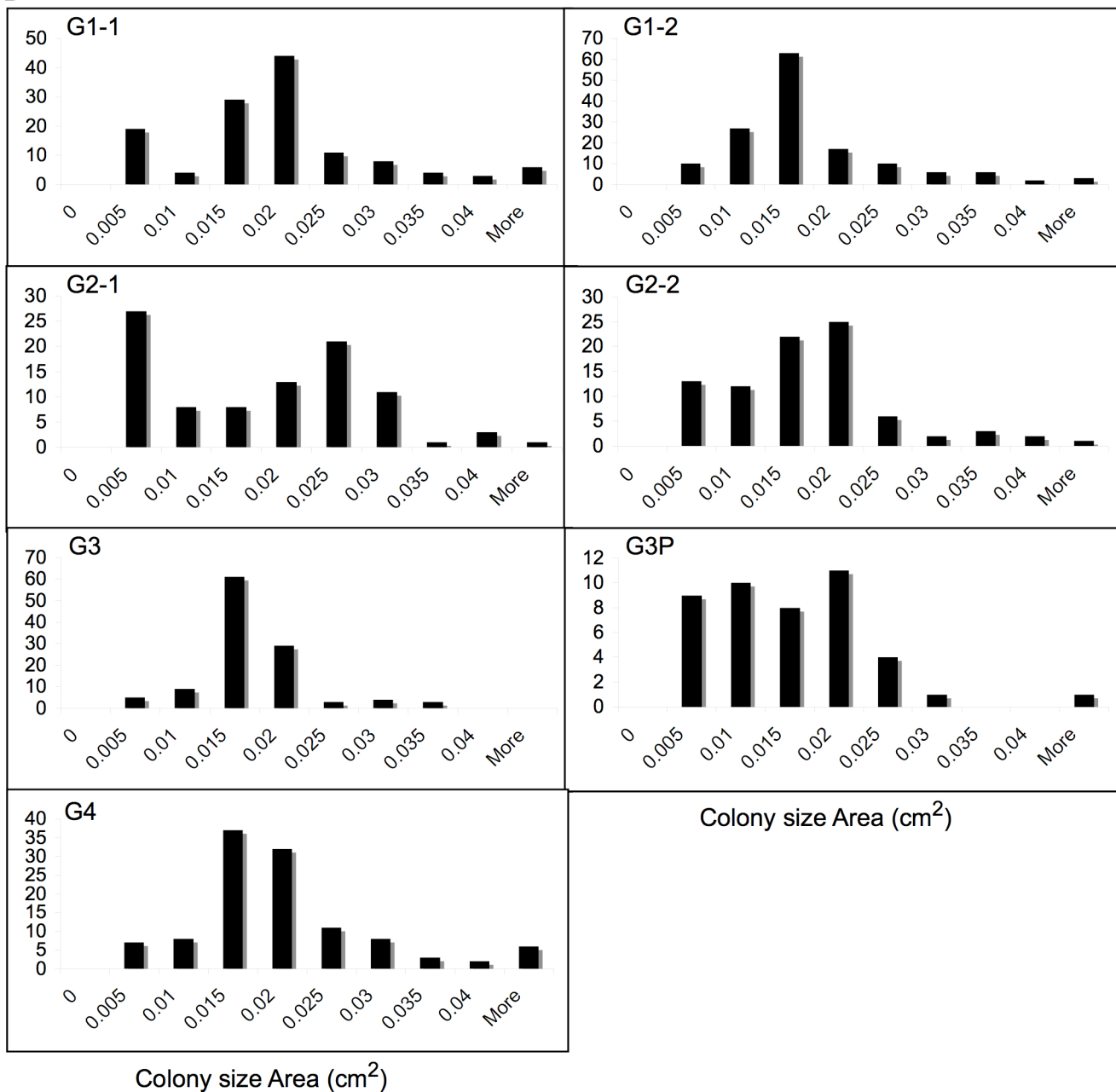

E

YPD

100mM HU

WT  
progenitor  
G1-1  
G1-2  
G2-1\*  
G2-2  
WT  
progenitor  
G3\*  
G3P\*  
G4

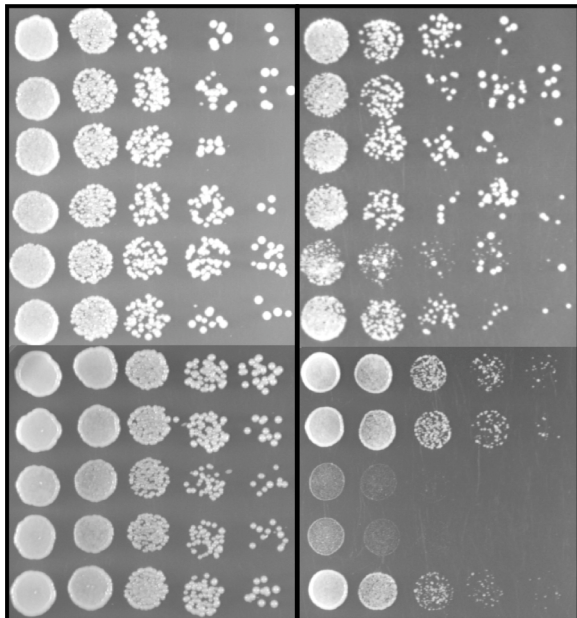

Supplement: Figure S2 — (2.84 MB PDF) [file pbio.1000161.s002.pdf]

# Common Gene loss on Chromosome IX in P4 and P6

Chromosome IX

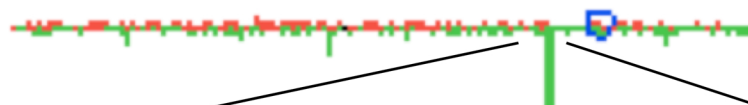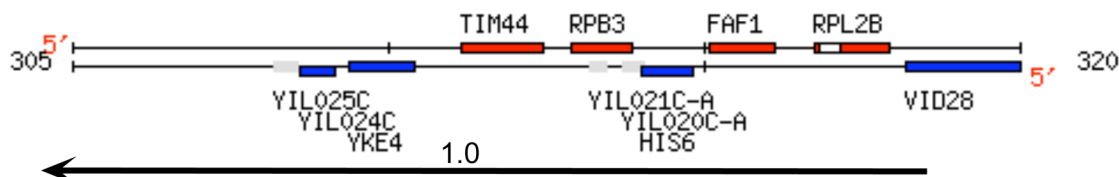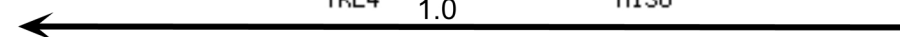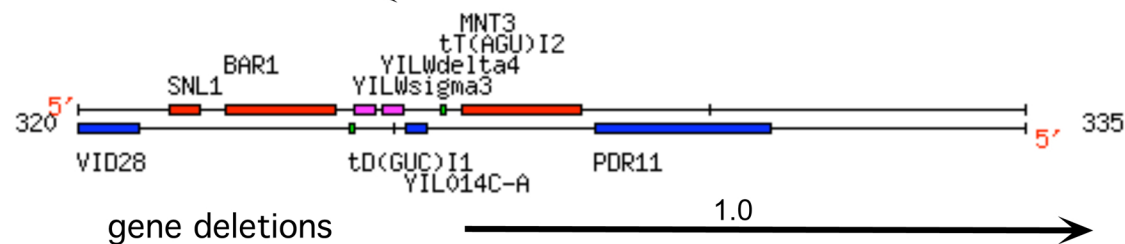

gene deletions

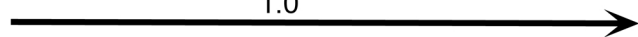

Supplement: Figure S5 — (0.55 MB PDF) [file pbio.1000161.s005.pdf]
