# Supplementary material for: Aneuploidy and Improved Growth Are Coincident but Not Causal in a Yeast Cancer Model
Source: PLoS Biol. 2009 Jul 28;7(7):e1000161. doi: 10.1371/journal.pbio.1000161 (PMC2708349; doi:10.1371/journal.pbio.1000161)

A Common Translocation between ChrVII and ChrXVI in G1-1 and G1-2

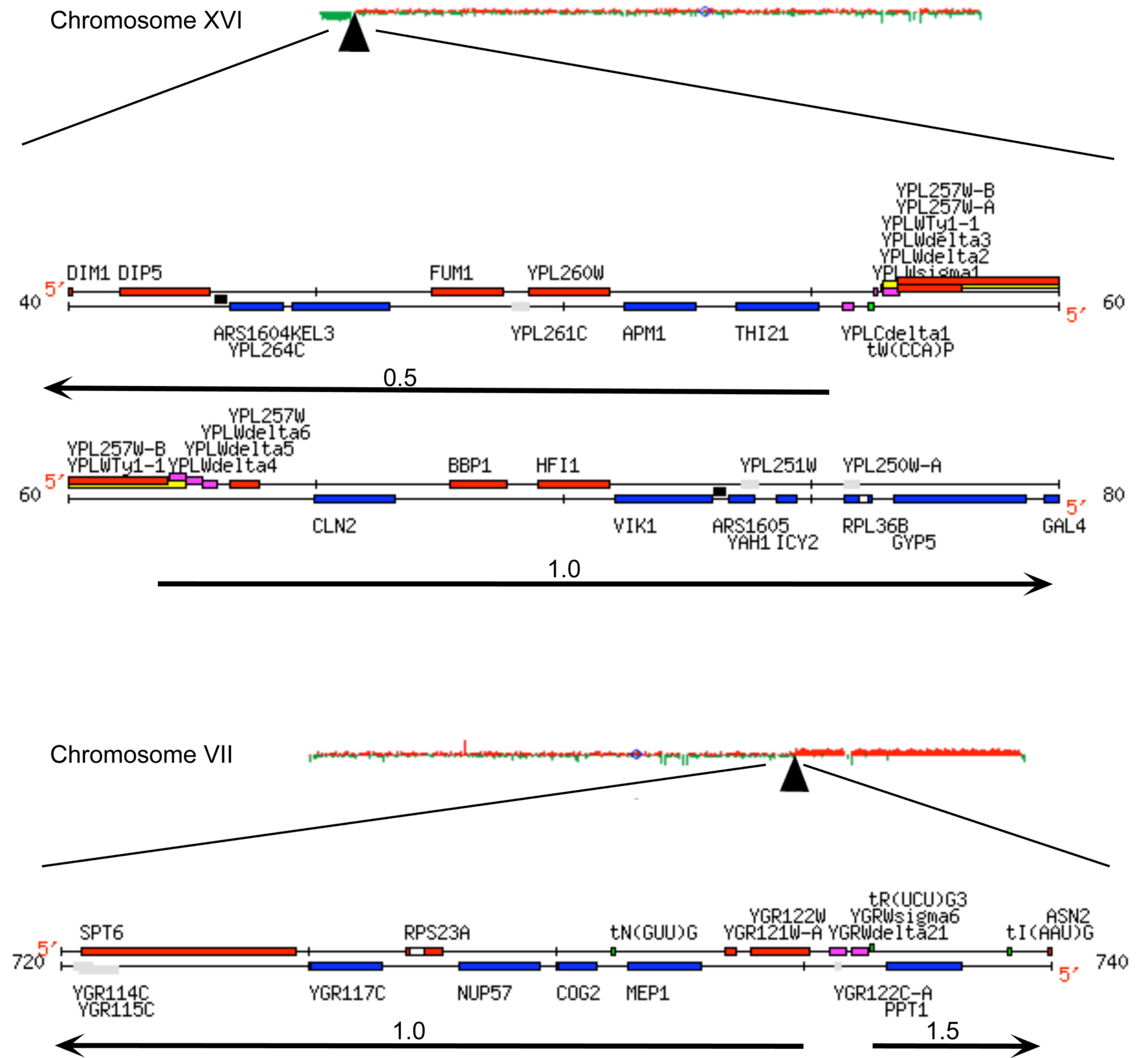

B Amplification in G2-2

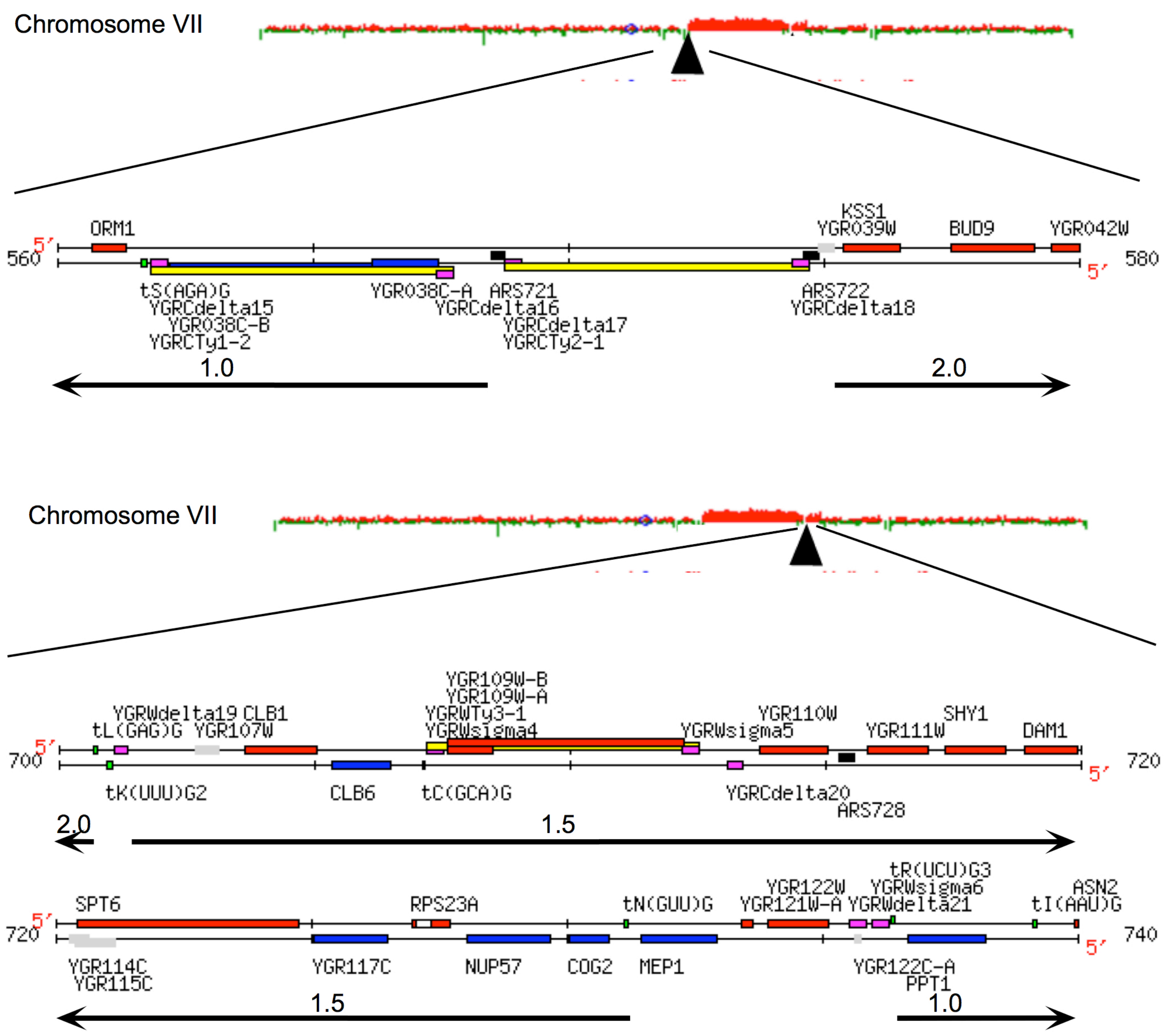

C translocation between chrVI and chrXII in G3 and G3P

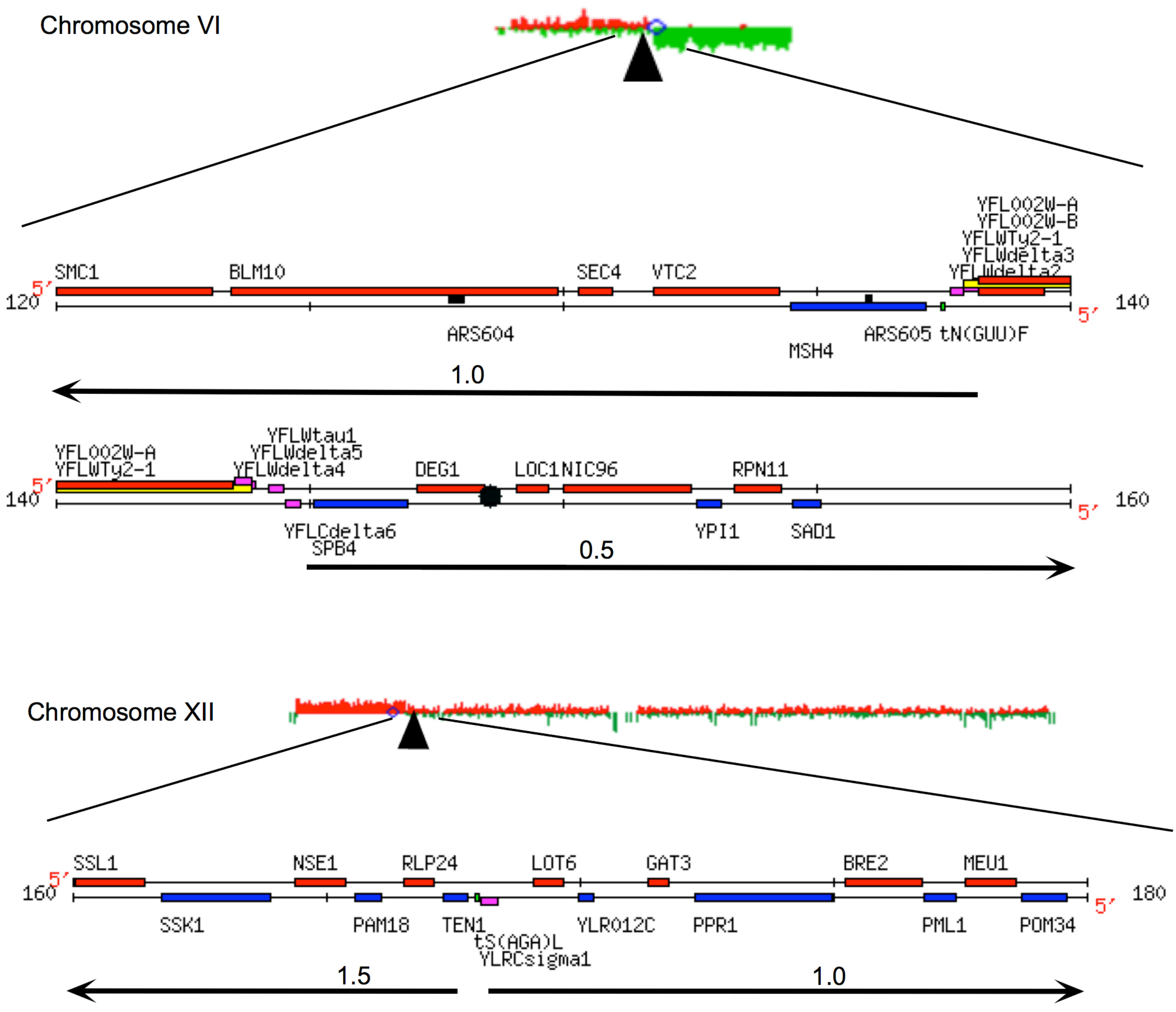

D Common intra-chromosomal deletion on chrV in G3, G3P and G4.

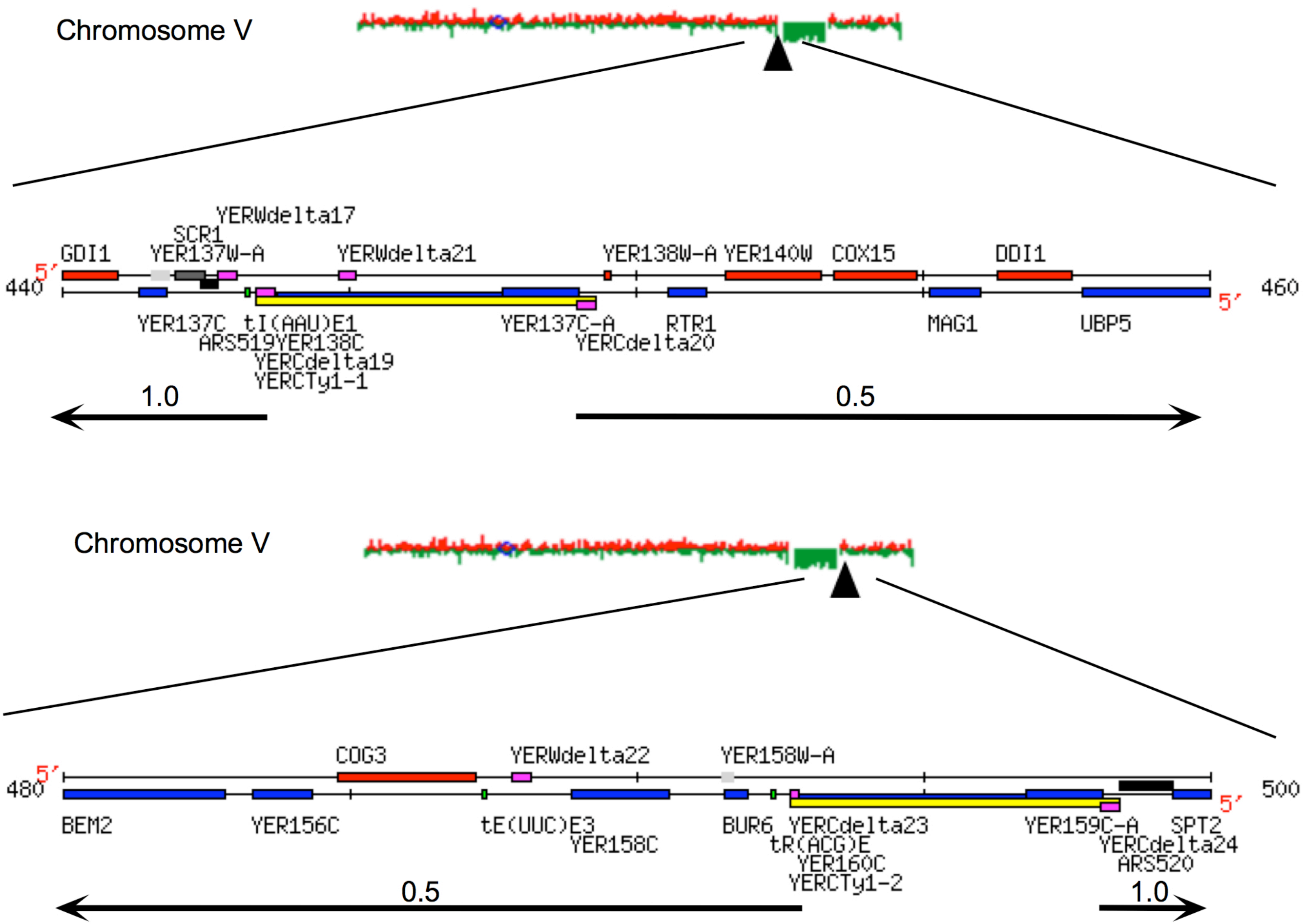

E Gene amplification on chrXIII in G4

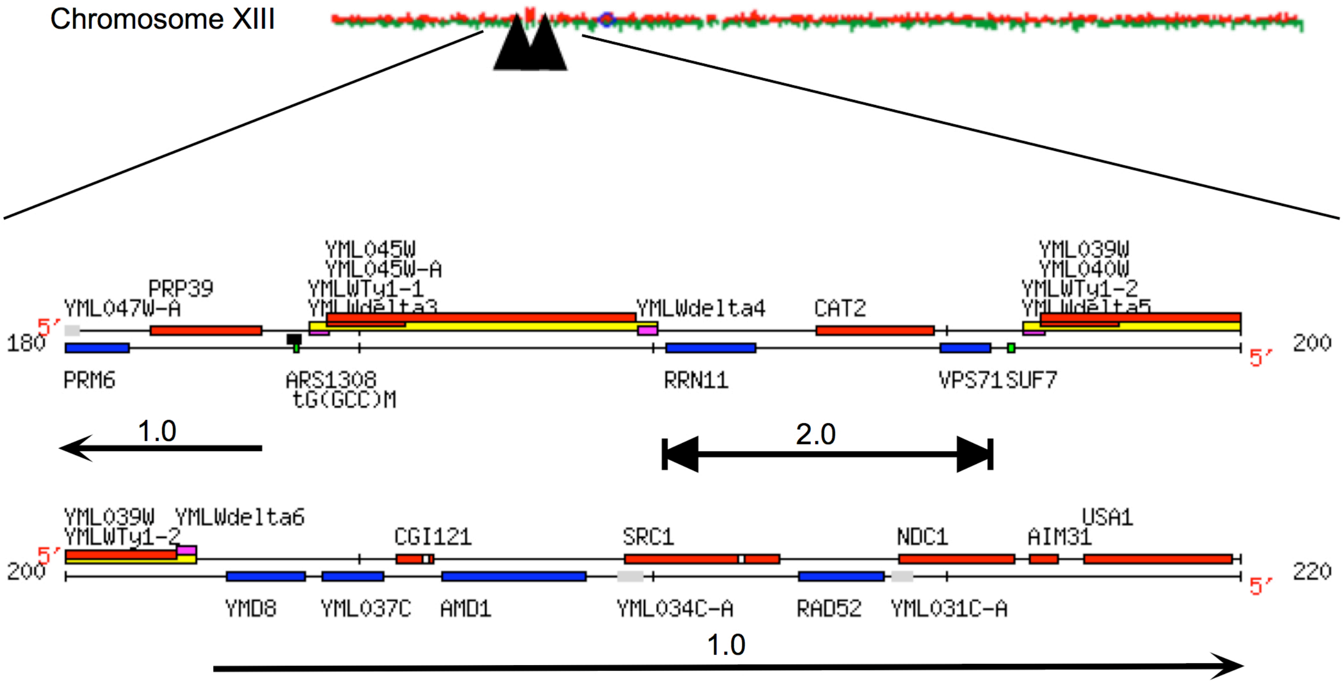

Supplement: Figure S3 — (4.33 MB PDF) [file pbio.1000161.s003.pdf]
