# Supplementary material for: Aneuploidy and Improved Growth Are Coincident but Not Causal in a Yeast Cancer Model
Source: PLoS Biol. 2009 Jul 28;7(7):e1000161. doi: 10.1371/journal.pbio.1000161 (PMC2708349; doi:10.1371/journal.pbio.1000161)
Supplement: Figure S4 — (3.31 MB PDF) [file pbio.1000161.s004.pdf]

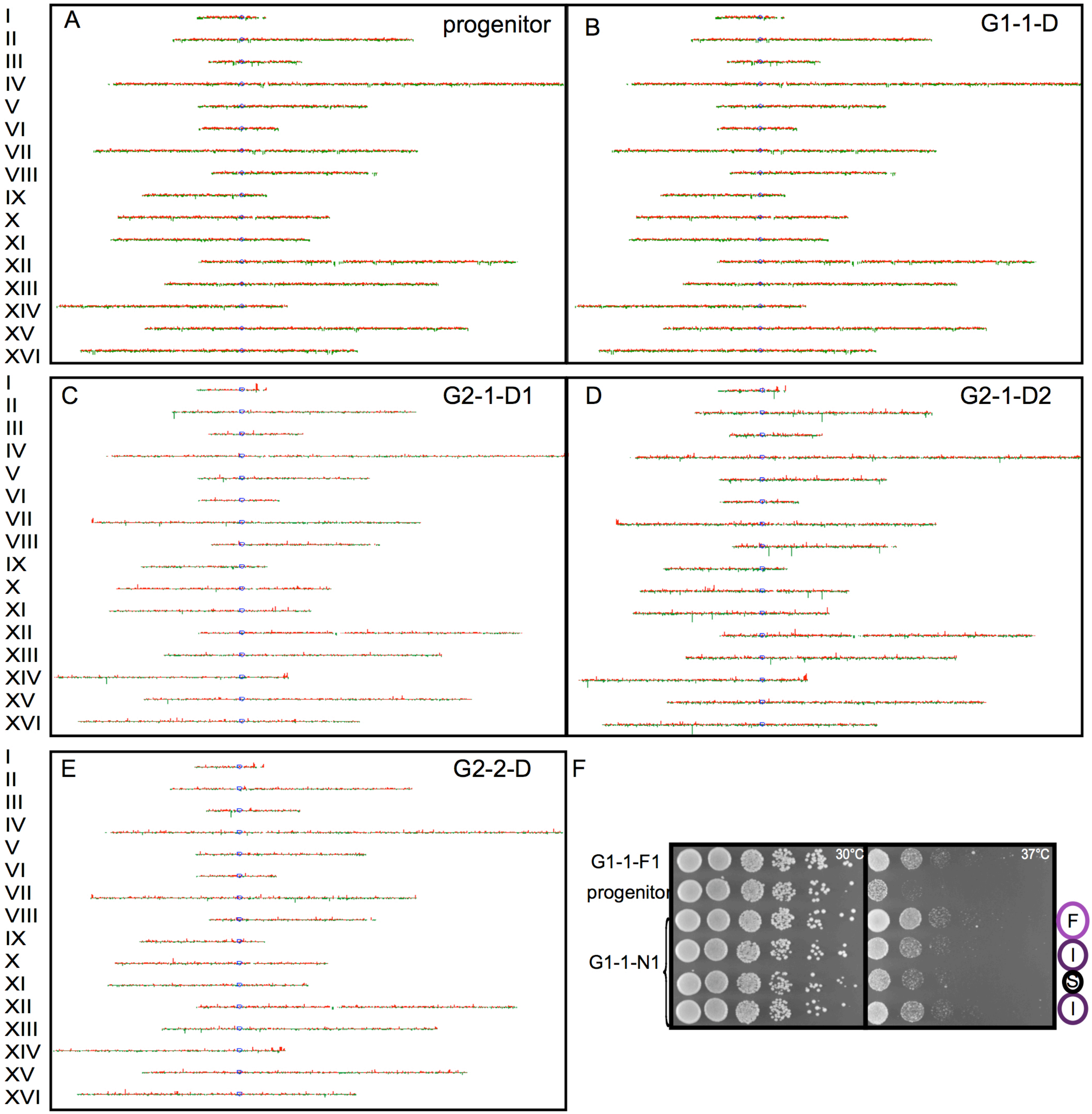
A
progenitor
B
G1-1-D
C
G2-1-D1
D
G2-1-D2
E
G2-2-D
F
I
II
III
IV
V
VI
VII
VIII
IX
X
XI
XII
XIII
XIV
XV
XVI
30°C
37°C
G1-1-F1
progenitor
G1-1-N1
F
I
S
I

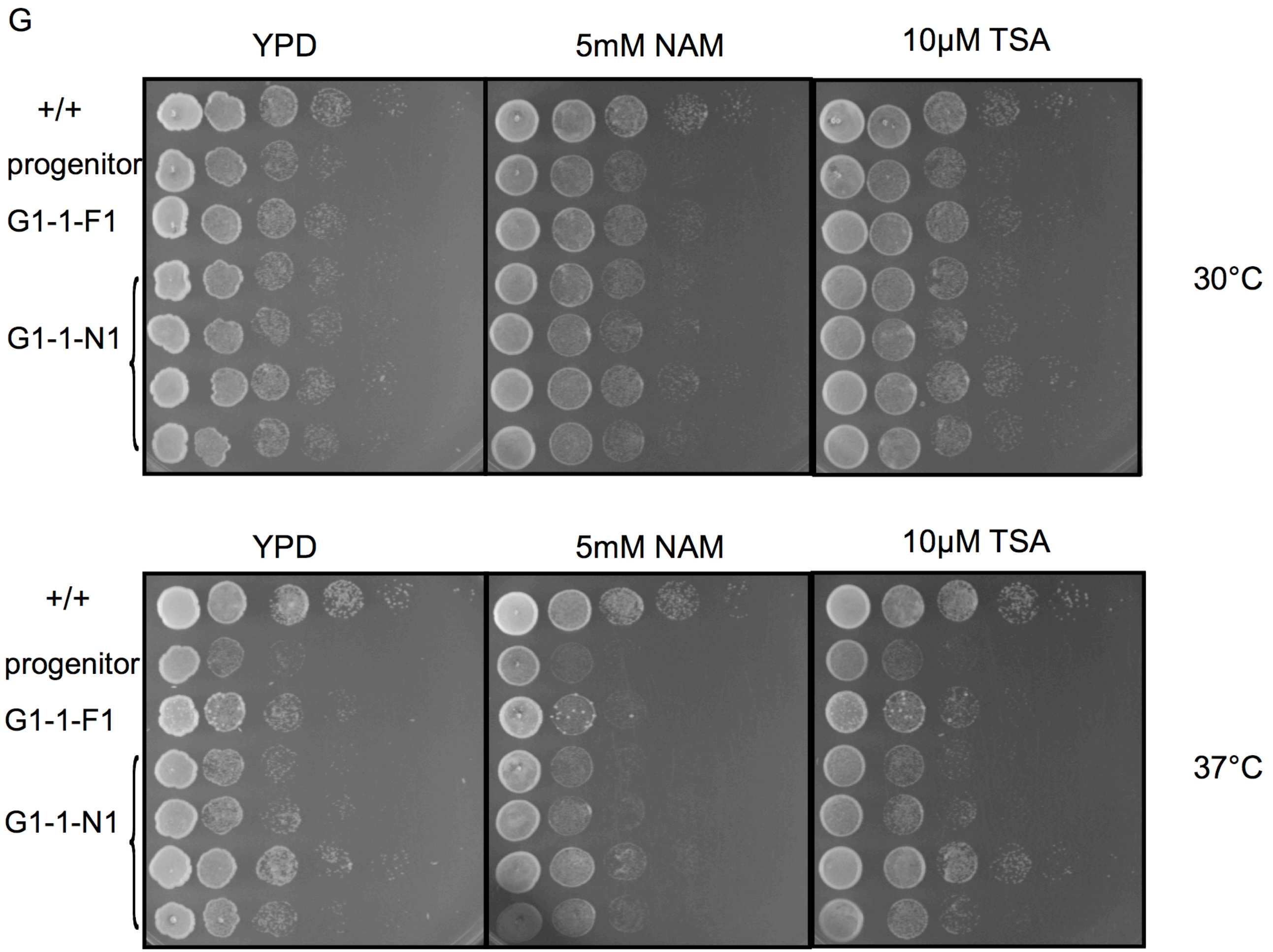
G
YPD
5mM NAM
10µM TSA
+/+
progenitor
G1-1-F1
G1-1-N1
30°C
YPD
5mM NAM
10µM TSA
+/+
progenitor
G1-1-F1
G1-1-N1
37°C
