# Supplementary material for: Aneuploidy and Improved Growth Are Coincident but Not Causal in a Yeast Cancer Model
Source: PLoS Biol. 2009 Jul 28;7(7):e1000161. doi: 10.1371/journal.pbio.1000161 (PMC2708349; doi:10.1371/journal.pbio.1000161)
Supplement: Table S1 — (0.07 MB DOC) [file pbio.1000161.s006.doc]

Table S1. Strain list

| **Strain** | **Genotype** | **Source** |
| --- | --- | --- |
| W303 (diploid) | MATa/MATα *ade2/ade2 his3/his3 leu2/leu2 trp1/trp1 ura3/ura3 can1/can1* |  |
| XLY495 | *mcm4^Chaos3/Chaos3^* | This lab |
| XLY494 | *mcm4^Chaos3/+^* | This lab |
| XLY506 | *mcm^+/Δ^* | This study |
| XLY507 | *mcm4^Chaos3/Δ^* | This study |
| XLY182 | *rad9:URA3/rad9:URA3* | Derived from strain 3834 from Judith Berman lab |
| XLY184 | *rad9:URA3/rad9:URA3 mcm4^Chaos3/Chaos3^* |
| XLY429 | *mad2:URA3/mad2:URA3* | Derived from RHC 15.1 from Kiwon Song lab |
| XLY431 | *mad2:URA3/mad2:URA3 mcm4^Chaos3/Chaos3^* |
| XLY270 | *hom3-10/HOM3 can1-100/CAN1* | Derived from MC42-2d and HLK1042-1C from Tom Petes lab |
| XLY462 | *hom3-10/HOM3 can1-100/CAN1 mcm4^Chaos3/+^* |
| XLY827 | *hom3-10/HOM3 can1-100/CAN1 mcm4^Chaos3/Chaos3^* |
| XLY385 | G1-1 *mcm4^Chaos3/Chaos3^* | This study |
| XLY499 | G1-2 *mcm4^Chaos3/Chaos3^* | This study |
| XLY386 | G2-1 *mcm4^Chaos3/Chaos3^* | This study |
| XLY500 | G2-2 *mcm4^Chaos3/Chaos3^* | This study |
| XLY496 | G3 *mcm4^Chaos3/Chaos3^* | This study |
| XLY502 | G3P *mcm4^Chaos3/Chaos3^* | This study |
| XLY510 | G4 *mcm4^Chaos3/Chaos3^* | This study |
| XLY516 | G1-1D *mcm4^Chaos3/Chaos3^* | This study |
| XLY536 | G2-1D1 *mcm4^Chaos3/Chaos3^* | This study |
| XLY537 | G2-1D2 *mcm4^Chaos3/Chaos3^* | This study |
| XLY534 | G2-2D *mcm4^Chaos3/Chaos3^* | This study |
| XLY593 | G1-1-W *mcm4^Chaos3/+^* | This study |
| XLY545 | G1-1F1 *mcm4^Chaos3/Chaos3^* | This study |
| XLY546 | G1-1F1’ *mcm4^Chaos3/Chaos3^* | This study |
| XLY543 | P4 *mcm4^Chaos3/Chaos3^* | This study |
| XLY544 | P6 *mcm4^Chaos3/Chaos3^* | This study |
| XLY715 | P6D *mcm4^Chaos3/Chaos3^* | This study |
| XLY723 | P6F1 *mcm4^Chaos3/Chaos3^* | This study |
| XLY719 | P6F2 *mcm4^Chaos3/Chaos3^* | This study |
